# Models of morality

**DOI:** 10.1016/j.tics.2013.06.005

**Published:** 2013-08

**Authors:** Molly J. Crockett

**Affiliations:** Wellcome Trust Centre for Neuroimaging, University College London, 12 Queen Square, London WC1N 3BG, UK

## Abstract

Moral dilemmas engender conflicts between two traditions: consequentialism, which evaluates actions based on their outcomes, and deontology, which evaluates actions themselves. These strikingly resemble two distinct decision-making architectures: a model-based system that selects actions based on inferences about their consequences; and a model-free system that selects actions based on their reinforcement history. Here, I consider how these systems, along with a Pavlovian system that responds reflexively to rewards and punishments, can illuminate puzzles in moral psychology.

## Consequentialist and deontological ethics

Is it morally permissible to kill one person to save five others? Moral dilemmas like this engender conflicts between two major traditions in normative ethics. Consequentialism judges the acceptability of actions based on their outcomes, and therefore supports killing one to save five; *ceteris paribus*, five lives are better than one. By contrast, deontology judges the acceptability of actions according to a set of rules; certain actions (e.g., killing) are absolutely wrong, regardless of the consequences.

Recent work has shown that experimental manipulations can sway people's judgments toward either consequentialism or deontology, suggesting that these perspectives have distinct neural underpinnings [1]. One influential account of these findings posits that deontological judgments stem from automatic emotional processes, whereas consequentialist judgments result from controlled cognitive processes [1]. Others argue that this dual-process approach is computationally insufficient and cannot explain how hypothetical scenarios are transformed into mental representations of actions and outcomes [2]. Universal moral grammar offers a computational theory of problem transformation, but lacks a neurobiologically plausible, mechanistic description of how values are assigned to mental representations of actions and outcomes, and how those values are integrated to produce a final consequentialist or deontological judgment.

## Model-based and model-free valuation

Recent advances in neuroscience offer a fresh perspective. Evaluations of actions and outcomes are guided by distinct decision-making systems that are psychologically and neurally dissociable 3, 4, 5, 6. The model-based system generates a forward-looking decision tree representing the contingencies between actions and outcomes, and the values of those outcomes. It evaluates actions by searching through the tree and determining which action sequences are likely to produce the best outcomes. Model-based tree search is computationally expensive, however, and can become intractable when decision trees are elaborately branched.

The computationally simple model-free system does not rely on a forward model. Instead, it evaluates actions based on their previously learned values in specific contexts (states): good state-action pairs are those that have produced desirable outcomes in the past (e.g., push door), whereas bad state-action pairs are those that have produced undesirable outcomes in the past (e.g., push person). Because the model-free system lacks access to current action–outcome links, it is retrospective rather than prospective and can make suboptimal recommendations in settings where traditionally good actions lead to undesirable outcomes, or *vice versa* 3, 6.

A third, Pavlovian system promotes automatic reflexive approach and withdrawal responses to appetitive and aversive stimuli, respectively [6]. Pavlovian biases can influence behaviors guided by model-based and model-free evaluations: for example, in aversive Pavlovian-to-instrumental transfer, aversive predictions can suppress instrumental actions [4]. Pavlovian biases can also influence model-based evaluations themselves: searching a decision tree can be conceptualized as a set of internal actions that can be suppressed by aversive predictions [6]. This amounts to a ‘pruning’ of the decision tree, whereby model-based tree search is curtailed when an aversive outcome is encountered [7].

There is now substantial evidence that model-based, model-free, and Pavlovian systems are situated in at least partly distinct brain circuits, although behavioral outputs likely reflect their combined influence 3, 5, and recent evidence suggests that certain regions integrate model-based and model-free evaluations [8]. These systems often arrive at similar conclusions about the best action to take, but they sometimes disagree. Understanding how such conflicts are resolved is an active topic of research [6].

## Models of morality

On the surface, consequentialism and deontology appear to map directly onto model-based and model-free systems, respectively. Consequentialist and model-based approaches both evaluate actions based on their outcomes, whereas deontological and model-free approaches both evaluate the actions themselves. However, a deeper analysis reveals that deontological judgments likely arise from sophisticated interactions between systems.

Consider one puzzle. In the classic trolley dilemma, a trolley is hurtling out of control down the tracks toward five workers, who will die if you do nothing. You and a large man are standing on a footbridge above the tracks. In one variant of this dilemma (trapdoor), you can flip a switch that will release a trapdoor, dropping the large man onto the tracks, where his body will stop the trolley. Is it morally permissible to flip the switch, killing the one man but saving the five workers? In another variant (push), you can push the large man off the footbridge onto the tracks, where again his body will stop the trolley. Is it morally permissible to push the man, killing him but saving the five workers? Intriguingly, when ordinary people confront these dilemmas, they are much less likely to endorse harming one to save five in cases in which harm involves physical contact with the victim (like pushing) than in cases in which harm does not involve physical contact (like releasing a trapdoor) 1, 2, even though these cases have identical outcomes.

Understanding how different decision systems evaluate actions and outcomes can illuminate puzzles such as the push–trapdoor divergence (Box 1). Consistent with universal moral grammar accounts [2], I propose that the model-based system transforms hypothetical scenarios into a structural description of actions and outcomes (i.e., a decision tree). By searching the tree, the model-based system evaluates all possible outcomes and recommends the action that leads to the best outcome.

Box 1 **Explaining aversion to physical harms**

Previous work attributes deontological judgments to automatic emotional processes [1]. Here, I distinguish between retrospectively rational (but re-trainable) model-free mechanisms and ecologically rational fixed Pavlovian mechanisms, both of which sway judgments toward deontology in cases in which harm involves physical contact.

The model-free system evaluates contextualized actions on the basis of their reinforcement history. Young children learn through experience that actions that physically harm others (e.g., hitting, pushing) result in aversive outcomes (e.g., punishments, distress cues 9, 10). Simultaneously, parents and society verbally instruct children that physical harm is forbidden, warning about the consequences of transgressions. Both experience and instruction enable the model-free system to attach negative value to harmful physical actions toward people, as in a class of algorithms (called Dyna) that complement experiential trial-and-error learning with hypothetical trial-and-error learning [6]. Importantly, the latter method, whereby model-free values can be retrained by model-based simulations, provides a route via which characteristically deontological judgments could be adaptive to changes in the environment that are detected by model-based mechanisms.

By contrast, the Pavlovian system triggers responses to predictions of valenced stimuli; whereas the values of stimuli can be learned, Pavlovian proclivities to approach (avoid) appetitive (aversive) stimuli are fixed, like reflexes. For aversive predictions, one type of Pavlovian response is behavioral suppression; this response is ecologically rational in the sense that refraining from action is generally a good strategy when some actions might produce aversive outcomes [4]. Aversive predictions embedded within moral dilemmas could evoke Pavlovian processes that disfavor active responses, leading to characteristically deontological judgments. Harmful actions that involve physical contact may generate particularly strong aversive predictions (e.g., fearful expressions, screams, gore).

Computational approaches to decision-making account for choices by adding up model-based, model-free, and Pavlovian action values, and then converting those values into action probabilities using a softmax function 4, 6, 7, essentially treating the three systems as separate experts, each of which ‘votes’ for its preferred action. Differences in state-action reinforcement histories (which influence model-free values) and aversive predictions (which influence Pavlovian values) could result in more ‘votes’ for inaction in the push scenario (Figure ID) than in the trapdoor scenario (Figure IC), leading to a higher proportion of deontological judgments in the former than in the latter.

**Figure I:**
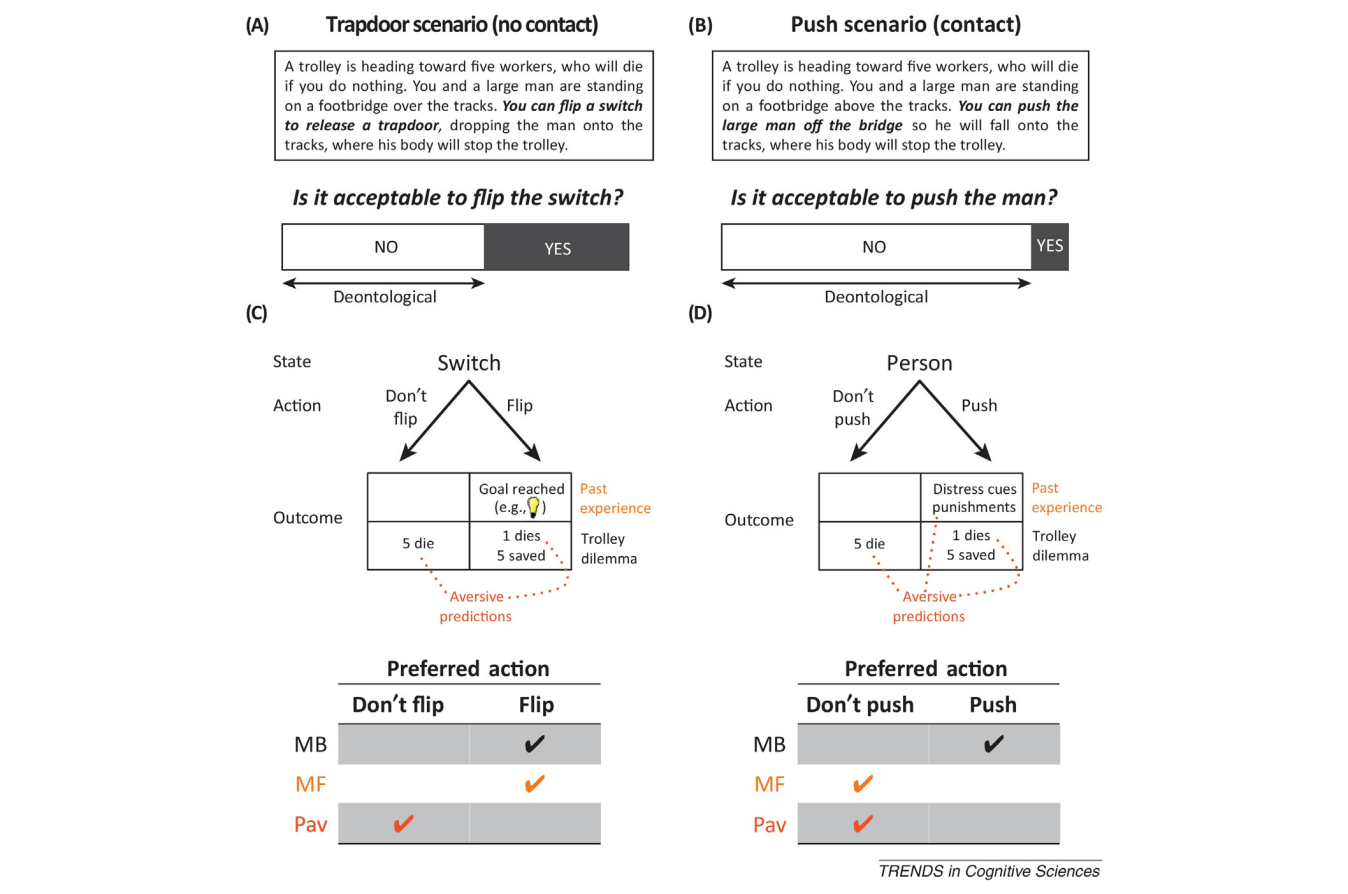
**(A,B)** Example trolley scenarios and typical judgment patterns (data adapted from [2]). **(C,D)** Diagrams mapping links between states, actions, and outcomes, and tables depicting the preferred actions of the model-based (MB), model-free (MF), and Pavlovian (Pav) systems.

Simultaneously, the model-free system evaluates contextualized actions, assigning negative values to state-action pairs with negative reinforcement histories (e.g., push person 9, 10). An important question is how the model-free system could evaluate actions that have never been performed directly (e.g., violent acts). One possibility is that action values are learned via observation: a recent study showed that observational model-free learning engages similar neural structures as does experiential model-free learning [11]. Alternatively, the model-based system could train the model-free system through off-line simulations [6].

Finally, the Pavlovian system may respond to model-based predictions of aversive outcomes (derived from the scenario text and represented in the decision tree) or, alternatively, to the model-free aversive values assigned to the described actions 4, 6. Each system ‘votes’ for its preferred action, and choices are a product of their combined influence. We can explain the difference in judgments for the push and trapdoor cases by considering that pushing a person and flipping a switch differ in terms of both their reinforcement histories and their proximal expected outcomes, which in turn influence the ‘votes’ of the model-free and Pavlovian systems (Box 1).

Consider a second feature of moral judgment: people readily distinguish between harm performed as a means to a desired end, and harm occurring as a foreseen side effect (Box 2). This distinction can be seen by contrasting the trapdoor case (described above) with the following side-track case: again, a trolley is hurtling out of control down the tracks toward five workers, who will die if you do nothing. You can flip a switch that will divert the trolley onto a different set of tracks, where a large man is standing. Is it morally permissible to flip the switch, killing the large man but saving the five workers? Despite the fact that outcomes are matched in these cases, people judge flipping the switch in the trapdoor case to be worse than in the side-track case. Why?

Box 2 **Explaining the means/side-effect distinction**

Building and searching through a decision tree can be conceptualized as a set of internal actions that may be susceptible to Pavlovian biases [6]. One example is the suppression of trains of thought that lead to aversive states, or a pruning of the decision tree [7]. Such pruning is Pavlovian in that it is reflexively evoked by aversive states and persists even when counterproductive, preventing the pursuit of rewards lurking behind aversive states [7].

A critical difference between means and side-effect cases is the position of harm within the decision tree, which has consequences for action evaluation in the face of pruning. Consider that model-based action evaluation integrates the outcomes from all branches of the decision tree, and Pavlovian pruning of branches containing aversive outcomes results in a reduced weighting of all the outcomes in a pruned branch. In side-effect cases, pruning results in a higher value of ‘flip switch’ by reducing the weight of the aversive outcome of killing one (compare the left and right panels in Figure IC). The side-effect death is incidental to saving the five individuals, so it can be safely pruned away while preserving the contribution of saving five toward the overall action value. This sort of selective amnesia for negative side effects is not possible in means cases, however. Here, because the aversive outcome of killing one is required to obtain the positive outcome of saving five, pruning away the aversive outcome necessarily also prunes away the positive outcome. Thus, in means cases, although pruning reduces the weight of the negative outcome of killing one (just as in side-effect cases), it also reduces the weight of the positive outcome of saving five (compare the left and right panels in Figure ID). The divergence in judgments between means and side-effect cases may be explained by the possibility that in the face of pruning, the positive outcome of saving five contributes less strongly to the overall action value in means cases (in which it is pruned away) compared to side-effect cases (in which it is not).

**Figure I:**
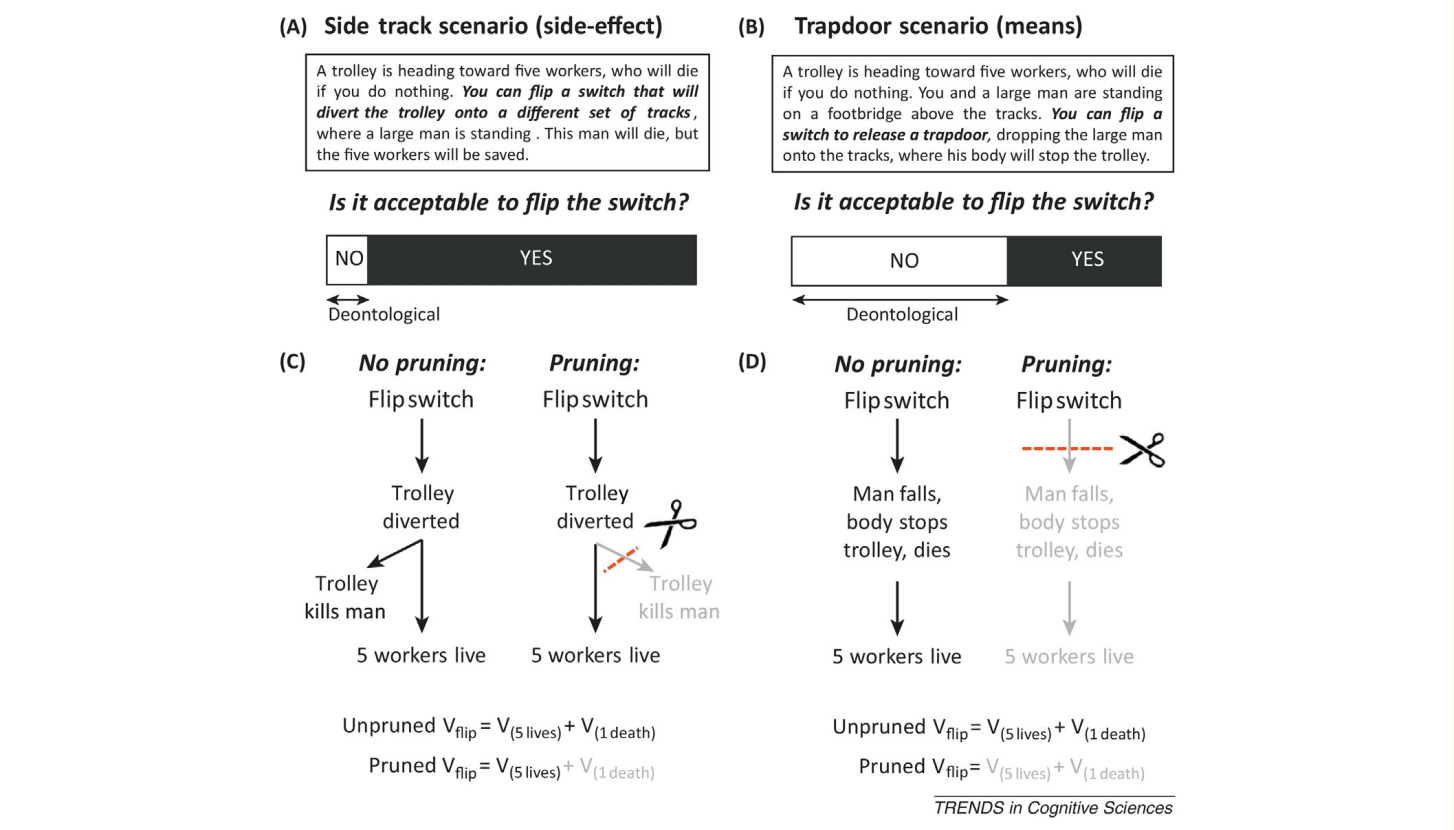
**(A,B)** Example trolley scenarios and typical judgment patterns (data adapted from [2]). **(C,D)** Decision trees representing actions and outcomes. Pruning occurs at the first aversive outcome encountered. The overall value for flipping the switch (*V*_flip_) is computed by adding the values from all tree branches: the positive value of saving five individuals (*V*_(5__lives)_) and the negative value of killing one (*V*_(1__death)_).

In the trapdoor case, the large man is used as a means: his body is instrumental in stopping the trolley, preventing it from hitting the five workers. In the side-track case, the death of the large man is a foreseen side-effect of the action performed to save the five workers. The structure of the decision tree is critical here; the means/side-effect distinction may arise from Pavlovian pruning of model-based tree search (Box 2) [7].

Because the neurobiology of model-based, model-free, and Pavlovian systems is reasonably well delineated, the current framework offers a parsimonious explanation of previous findings. For example, the medial prefrontal cortex (mPFC) seems to play a role in integrating model-free and model-based evaluations 3, 5. The proposal that model-free evaluations contribute to deontological judgments, together with the possibility that the mPFC incorporates model-free values into moral judgments, can account for two robust findings in the literature: that physical contact cases such as push, commonly associated with deontological judgments, activate the mPFC [1]; and that patients with mPFC lesions show a reduced tendency toward deontological judgments in those same cases [12]. Pavlovian aversive predictions have been linked to serotonin function 4, 7; if such predictions play a key role in deontological judgments, enhancing serotonin function should increase deontological judgments, which has indeed been demonstrated [13]. Finally, there is evidence that stress shifts control from model-based to model-free systems [14]. My account suggests that stress should similarly promote deontological judgments, as recently reported [15].

## Concluding remarks

One benefit of multiple decision systems is that each provides advantages in certain situations. Model-based control is optimal for simple decisions. However, when the decision tree is too extensive and tree search becomes computationally intractable, model-free and Pavlovian mechanisms provide useful heuristics. Perhaps multiple ethical systems exist for similar reasons. Consequentialism provides a flexible framework for maximizing good and minimizing evil, but in the face of uncertain or ambiguous outcomes that are commonly encountered in real-world moral dilemmas, deontological rules help to keep us out of trouble. Whether normative models of decision-making can inform normative ethics remains a tantalizing question.

## References

[1] Greene J.D., Gazzaniga M. (2009). The cognitive neuroscience of moral judgment. The Cognitive Neurosciences.

[2] Mikhail J. (2007). Universal moral grammar: theory, evidence and the future. Trends Cogn. Sci..

[3] Balleine B.W., O’Doherty J.P. (2009). Human and rodent homologies in action control: corticostriatal determinants of goal-directed and habitual action. Neuropsychopharmacology.

[4] Huys Q.J.M. (2011). Disentangling the roles of approach, activation and valence in instrumental and Pavlovian responding. PLoS Comput. Biol..

[5] Wunderlich K. (2012). Mapping value based planning and extensively trained choice in the human brain. Nat. Neurosci..

[6] Dayan P. (2012). How to set the switches on this thing. Curr. Opin. Neurobiol..

[7] Huys Q.J.M. (2012). Bonsai trees in your head: how the Pavlovian system sculpts goal-directed choices by pruning decision trees. PLoS Comput. Biol..

[8] Daw N.D. (2011). Model-based influences on humans’ choices and striatal prediction errors. Neuron.

[9] Cushman F. (2012). Simulating murder: the aversion to harmful action. Emotion.

[10] Blair R. (1995). A cognitive developmental approach to morality: investigating the psychopath. Cognition.

[11] Liljeholm M. (2012). Dissociable brain systems mediate vicarious learning of stimulus–response and action–outcome contingencies. J. Neurosci..

[12] Koenigs M. (2007). Damage to the prefrontal cortex increases utilitarian moral judgements. Nature.

[13] Crockett M.J. (2010). Serotonin selectively influences moral judgment and behavior through effects on harm aversion. Proc. Natl. Acad. Sci. U.S.A.

[14] Schwabe L., Wolf O.T. (2013). Stress and multiple memory systems: from ‘thinking’ to ‘doing’. Trends Cogn. Sci..

[15] Youssef F.F. (2012). Stress alters personal moral decision making. Psychoneuroendocrinology.

